# Supplementary material for: Assessing respiratory epidemic potential in French hospitals through collection of close contact data (April–June 2020)
Source: Sci Rep. 2024 Feb 14;14:3702. doi: 10.1038/s41598-023-50228-8 (PMC10866902; doi:10.1038/s41598-023-50228-8)
Supplement: Supplementary file 1 — Supplementary Information. [file 41598_2023_50228_MOESM1_ESM.pdf]

Supplementary Material

EMAE-MESuRS Working Group on Nosocomial SARS-CoV-2 Modelling

George Shirreff[1,2,3], Audrey Duval[1], Laura Temime[3,24], Lulla Opatowski[1,2], Sophie Chervet[1,2], Kévin Jean[3,24,26], Sofía Jijón[3,24], Ajmal Oodally[1,2,3], David RM Smith[1,2,3], Niels Hendrickx[1], Kenza Hamzi[3] and Cynthia Tamandjou[1].

[1]Institut Pasteur, Université Paris Cité, Epidemiology and Modelling of Antibiotic Evasion; Paris, France
[2]Université Paris-Saclay, UVSQ, Inserm, CESP, Anti-infective evasion and pharmacoepidemiology team ; Montigny-Le-Bretonneux, France
[3]Modélisation, épidémiologie et surveillance des risques sanitaires (MESuRS), Conservatoire National des Arts et Métiers; Paris, France
[24]PACRI unit, Institut Pasteur, Conservatoire national des Arts et Métiers; Paris, France
[26]MRC Centre for Global Infectious Disease Analysis, Department of Infectious Disease Epidemiology, Imperial College London, London, UK

Supplementary Figures

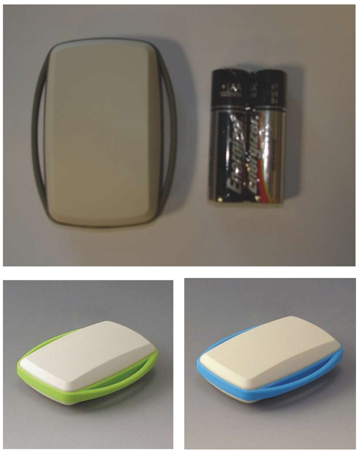

Supplementary Figure S 1. ***Wearable sensor for measurement of close proximity interactions.*** *WSN430 wearable sensor, about 4cm x 6cm. Sensors are powered by a TI MSP430 micro-controller and communicates through a TI Chipcon CC1100 packet radio interface, with a memory and a battery. Sensors were kept in a low-power mode (sleep mode) for most of the time, and awakened every 10 seconds to broadcast information (a "Hello" packet). When a sensor received this from another sensor, it logged that interaction and the time. Data from the sensor was uploaded upon its return.*

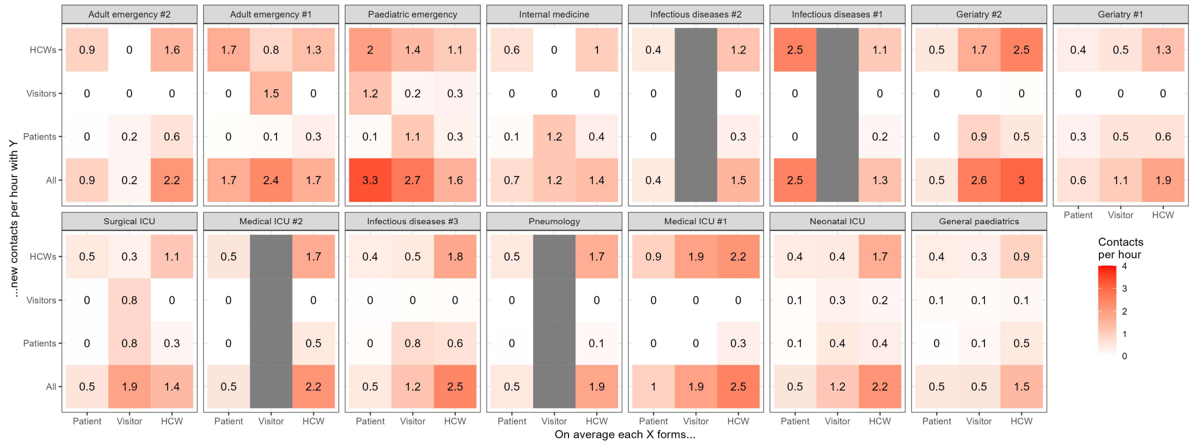

Supplementary Figure S 2. **Contact rate between types of individuals on each ward.** Each panel represents a ward, and each cell represents the contact rate that each status of individual (patient, visitor or HCW, columns) has with each type of individual (rows). Contact rate is the number of unique new contacts formed throughout the study period, per hour spent carrying the sensor. Where the type of individual is not present, the corresponding column is is grey.

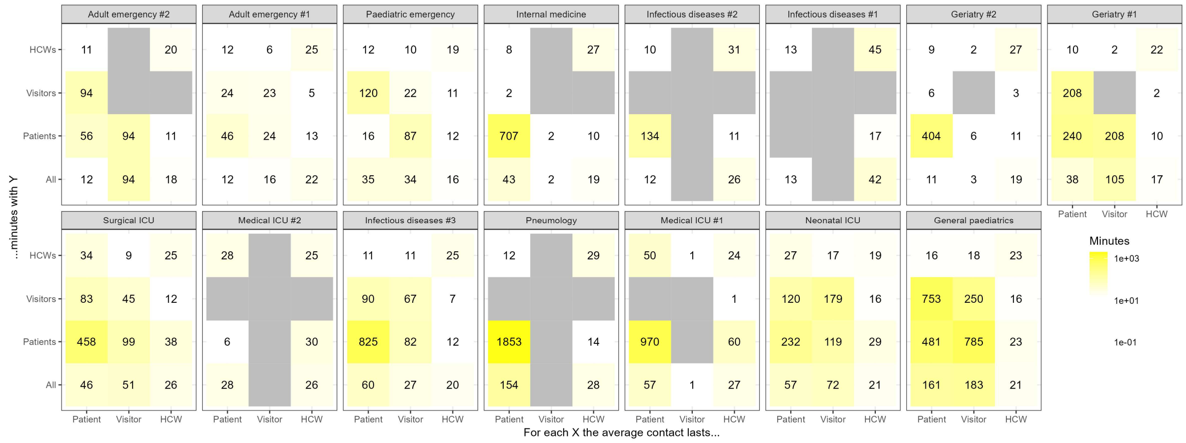

Supplementary Figure S 3. **Duration of contacts between types of individuals on each ward.** Each panel represents a ward, and each cell represents the average contact duration that each type of individual (patient, visitor or HCW, columns) has with an individual of each type (rows). Average contact duration is calculated for each individual with status Y, before the average for the column is calculated across all individuals of status X. Where the individual in the row and column have no contact, the average duration of contact is not calculable and the cell is grey.

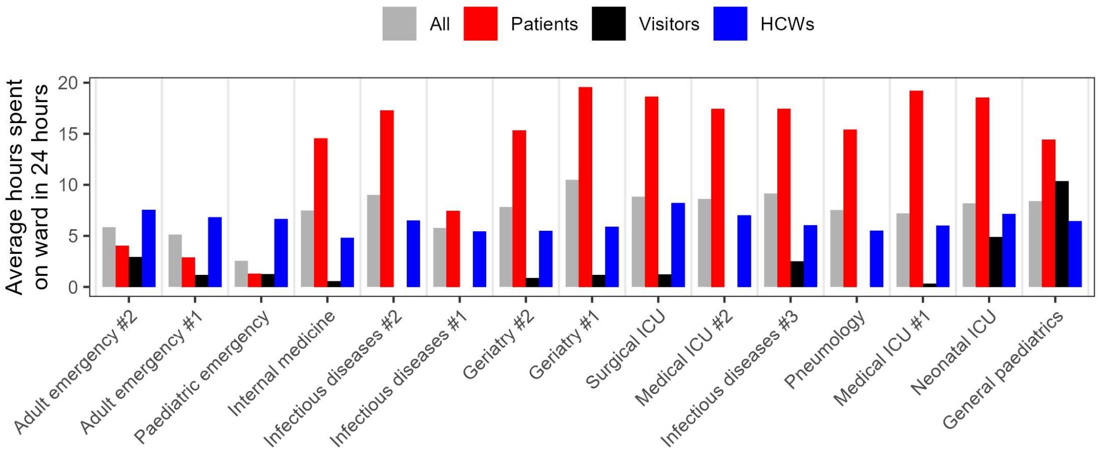

Supplementary Figure S 4. ***Average amount of time spent on the ward (carrying the sensor) by each status of individual, normalised to 24 hours.***

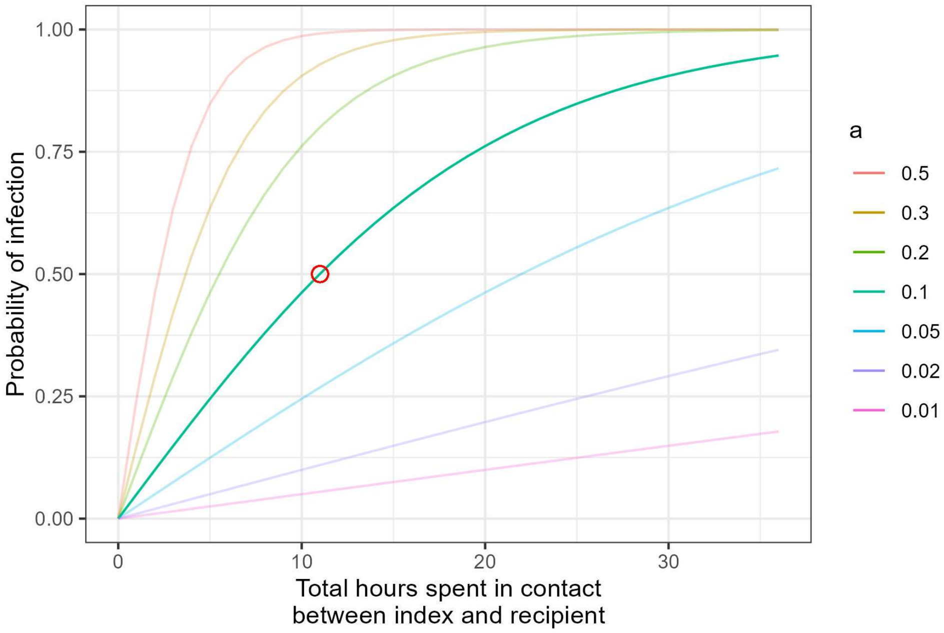

Supplementary Figure S 5. ***Relationship between duration of contact and probability of infection.*** *For different values of a, the probability of infection during a contact of duration hours between an infectious and susceptible individual, according to Equation [7]. The value a=0.1 is used for the baseline analysis, and for illustration, the time at which probability of infection is 50% is marked with a red circle.*

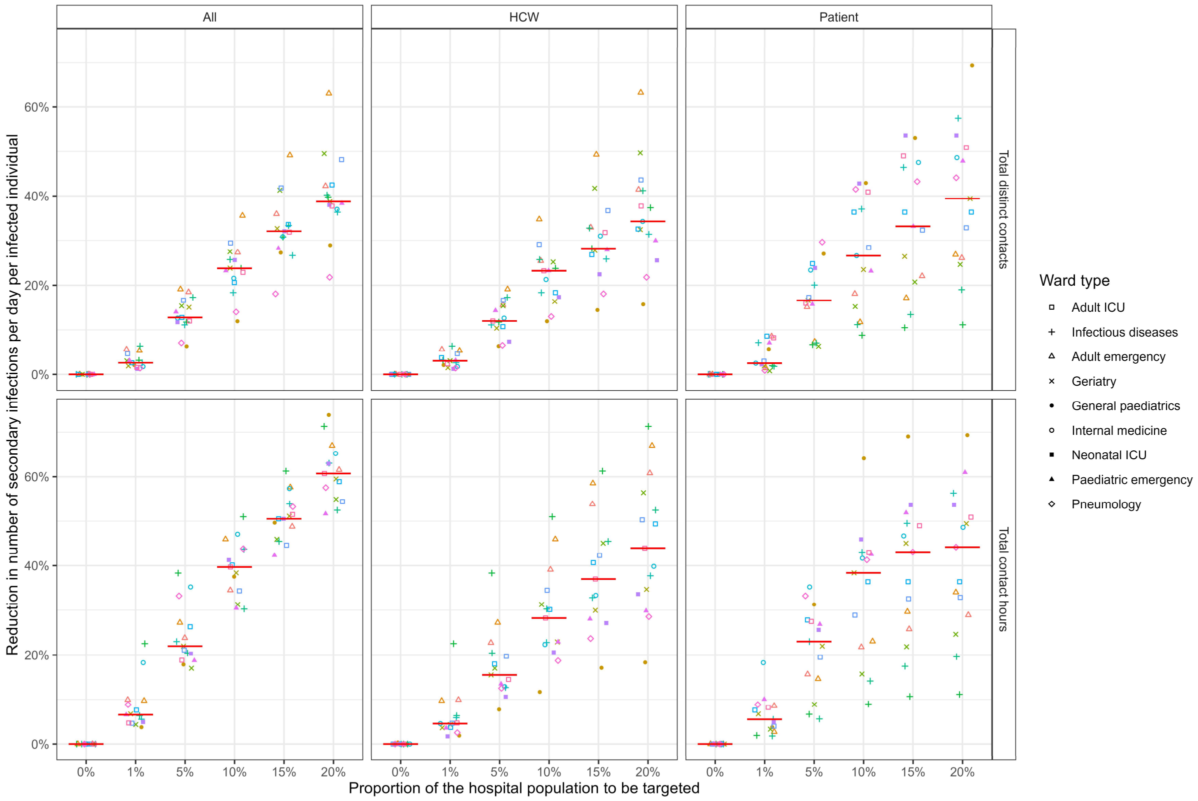

Supplementary Figure S 6. ***The percentage reduction in number of secondary infections per day per infected individual when the most connected X% part of the population are completely protected.*** *In each panel, the indicated proportion is taken from the group indicated by the panel column. If the proportion to be targeted is larger than the proportion of that group present, the entire group is excluded. The x-axis indicates the proportion protected. Each point represents a single ward, and the horizontal red line represents the median across all wards.*

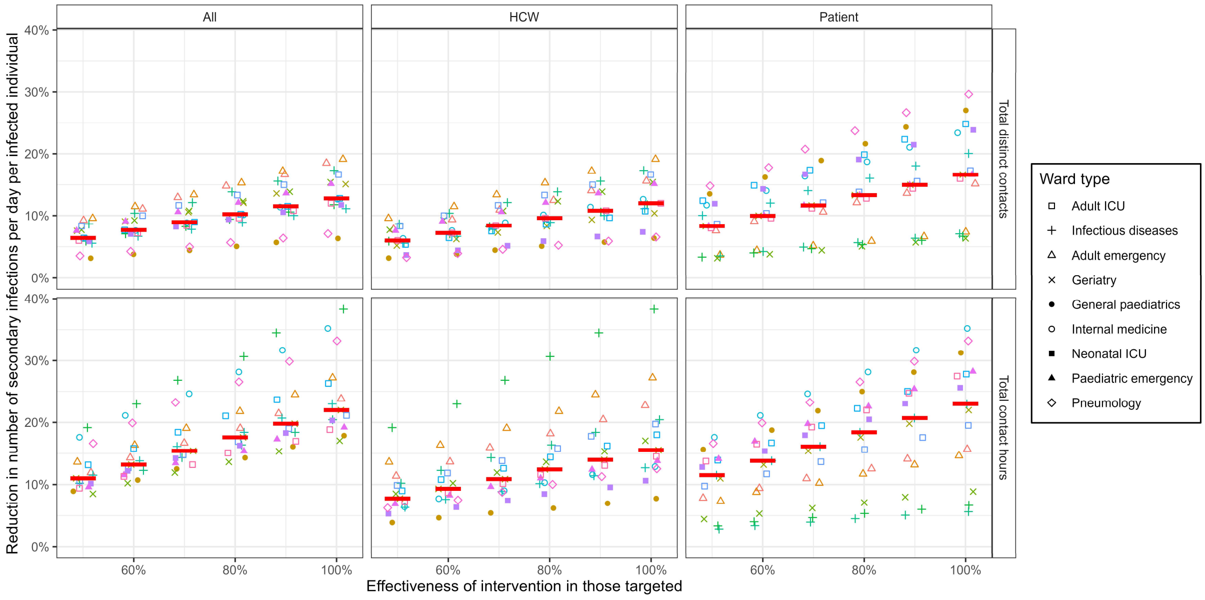

Supplementary Figure S 7. ***The percentage reduction in number of secondary infections per day per infected individual (y-axis) at different levels of intervention efficacy (x-axis).*** *In each panel, the intervention is targeted to 5% of the population, taken only from the group in the panel column, and using either their total distinct contacts (top row) or total contact hours (bottom row).*

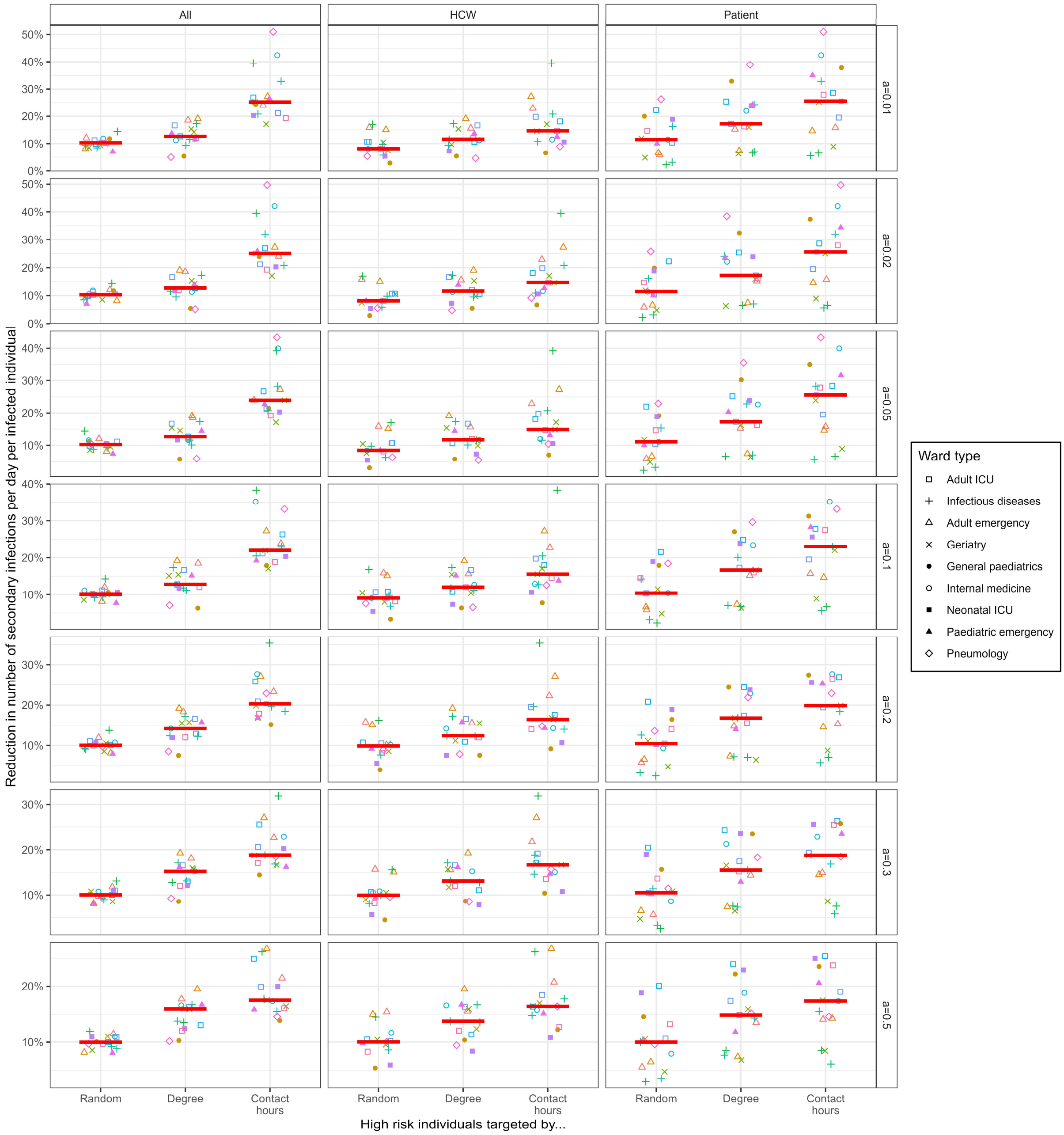

Supplementary Figure S 8. ***The percentage reduction in risk for different values of the shape parameter* a.** *For different values of the shape parameter* a *(rows of panels), the percentage reduction in risk is calculated when the most connected 5% of the population are completely protected. In each panel, the 5% are taken only from group indicated on the column. The x-axis indicates the targeting method. The horizontal red line indicates the median across all wards.*

Supplementary Tables

Supplementary Table S 1. ***List of wards investigated.*** *Regional weekly hospitalization was the 7-day moving average of hospital admissions with SARS-CoV-2 in the corresponding region on the first day of the study (Sante Publique France, tx_indic_7J_hosp). The totals refer to the number of HCW, patients and visitors who had contact data recorded during the study. The peak hospital users is the most number of these individuals who were present at any one time.*

Medical specialty	Date of investigation	Regional weekly hospitalisation	Peak hospital users	Total			
				HCW	Patients	Visitors	All
Infectious diseases #1	2020, April 17-18	33.12	45	66	14	0	80
Pneumology	2020, April 20-21	28.61	60	99	25	0	124
Internal medicine	2020, April 24-25	21.09	39	51	20	1	72
Infectious diseases #2	2020, May 4-5	10.30	50	66	20	0	86
Medical ICU #1	2020, May 6-7	9.18	88	175	18	1	194
General paediatrics	2020, May 13-14	6.35	53	66	16	18	100
Geriatry #1	2020, May 18-19	5.32	82	80	41	1	122
Paediatric emergency	2020, May 21-22	3.96	64	80	126	128	334
Neonatal ICU	2020, May 25-26	3.22	69	81	18	31	130
Geriatry #2	2020, June 4-5	0.76	84	109	36	3	148
Medical ICU #2	2020, June 9-10	0.54	61	95	17	0	112
Adult emergency #1	2020, June 11-12	0.50	51	96	61	6	163
Surgical ICU	2020, June 16-17	0.60	64	74	18	17	109
Adult emergency #2	2020, June 18-19	0.56	72	101	92	1	194
Infectious diseases #3	2020, June 23-24	0.07	98	99	52	19	170

Supplementary Table S 2. ***Population description by category.*** *Total approached, the number (and proportion) who participated, who refused, for whom the captor was lost (and data therefore unrecorded), and the mean age of participants.*

Category	Total	Participated	Refused	Lost	Mean age (range)
Nurse	359	347 (97%)	6 (2%)	6 (2%)	34 (22-64)
Physician	305	277 (91%)	4 (1%)	24 (8%)	36 (24-68)
Auxilliary nurse	280	262 (94%)	11 (4%)	7 (3%)	38 (20-62)
Other	124	105 (85%)	2 (2%)	17 (14%)	39 (19-64)
Administratitive	98	89 (91%)	2 (2%)	7 (7%)	46 (23-67)
Student physician	92	86 (93%)	2 (2%)	4 (4%)	24 (21-52)
Logistic	109	69 (63%)	5 (5%)	35 (32%)	38 (19-63)
Student nurse	47	46 (98%)	0	1 (2%)	26 (19-51)
Reeducation	39	39 (100%)	0	0	32 (22-57)
Children (0-18)	239	160 (67%)	49 (21%)	30 (13%)	6 (0-18)
Adults	413	413 (100%)	0	0	65 (19-103)
Visitor	280	221 (79%)	17 (6%)	42 (15%)	41 (0-88)
Total	2385	2114 (89%)	98 (4%)	173 (7%)	40 (0-103)
